# Coffee intake leads to preeclampsia-like syndromes in susceptible pregnant rats

**DOI:** 10.1017/jns.2024.36

**Published:** 2024-09-18

**Authors:** Linyan Chen, Yi Duan, Pan Wang

**Affiliations:** Shenzhen Key Laboratory of Steroid Drug Discovery and Development, School of Medicine, The Chinese University of Hong Kong, Shenzhen, Guangdong, China

**Keywords:** Coffee, Fetus, Preeclampsia, Pregnancy

## Abstract

Coffee is one of the most popular beverages worldwide, and there is an increasing concern of the health risk of coffee consumption in pregnancy. Preeclampsia (PE) is a serious pregnancy disease that causes elevated blood pressure and proteinuria in pregnant women and growth restriction of fetuses due to poorly developed placental vasculature. The aim of our study is to investigate the possible effect of coffee intake during pregnancy in rats with potential underlying vasculature conditions. The endothelial nitric oxide synthase inhibitor N(gamma)-nitro-L-arginine methyl ester (L-NAME) at a high dose (125 mg/kg/d) was used to induce PE in pregnant rats, which were used as the positive control group. In addition, low-dose L-NAME (10 mg/kg/d) was used to simulate the compromised placental vasculature function in pregnant rats. Coffee was given together with low-dose L-NAME to the pregnant rats from gestational day 10.5–18.5. Our results show that the pregnant rats treated with low-dose L-NAME + coffee, but not low-dose L-NAME alone, developed PE symptoms such as prominent fetal growth restriction, hypertension, and proteinuria. Therefore, our findings suggest that coffee intake during pregnancy may cause an increased risk of PE in susceptible women.

## Introduction

Preeclampsia (PE) is a pregnancy-specific disease with hypertension as the main maternal symptoms during the latter half of pregnancy and fetal growth restriction.^(1)^ The mechanisms of PE include deficient trophoblast invasion and incomplete spiral artery remodelling in the placenta.^(1)^ The incidence of PE has increased recently with about eight million new cases per year.^(2,3)^ However, the causes of PE remain elusive, which makes it difficult to prevent the condition.^(4,5)^

Coffee, a popular beverage worldwide, contains numerous dietary compounds such as tannic acid, chlorogenic acid, *etc.*, which are generally thought to be beneficial for health.^(6,7)^ However, coffee consumption during pregnancy, has been found to be associated with an increased risk of stillbirth, fetal abnormalities, and miscarriage as reported by several epidemiological studies.^(8,9)^ These findings indicate that coffee consumption during pregnancy may have potential hazardous effect.

N(gamma)-nitro-L-arginine methyl ester (L-NAME) is an inhibitor of nitric oxide synthase (NOS), and L-NAME is one of the commonly used compounds to induce PE in experimental animals.^(10,11)^ The reason is that endothelial nitric oxide synthase (eNOS) plays an important role in protecting blood vessels from hypoxia and ischemia, which are major causes of hypertension in PE.^(10)^ Therefore, in this study, L-NAME (125 mg/kg per d)-treated Sprague-Dawley (SD) rats were used as the positive control group for inducing PE. This dose is similar to that used in the literature.^(10)^ In addition, we used low-dose L-NAME (10 mg/kg per d) treatment in pregnant rats to simulate the pregnancy with predisposed underlying vascular condition caused by reduced NOS activity, and then we studied whether coffee could induce PE in these low-dose L-NAME treated rats. The results of this study provide experimental evidences on the potential health hazards of coffee intake during pregnancy.

## Materials and methods

### Chemicals and reagents

Sinloy Coffee powder (Caffeine content 1.0–1.5%) was purchased from Baoshan Zhongka Food Co. Ltd (Baoshan, Yunnan, China). L-NAME was obtained from Macklin (Beijing, China). The enzyme-linked immunosorbent assay (ELISA) kit for soluble fms-like tyrosine kinase-1 (sFlt-1) was purchased from QSBIO (China). The kits for determination of urinary albumin, creatinine, serum uric acid were obtained from Nanjing Jiancheng Bioengineering Institute (Nanjing, China).

### Animal experiment

All the animal experiment details were approved by the Institutional Animal Care and Use Committee of the Chinese University of Hong Kong (Shenzhen) (the approval number is CUHKSZ-AE2021004) according to the national guidelines for the care and use of experimental animals and ARRIVE Guidelines. All animals were raised in the specific pathogen free environment with a light/dark cycle of 12 h, and given free access to food and water. SD rats were obtained from Vital River Laboratory Animal Technology (Beijing, China). Virgin female rats (12–14 weeks, with body weight 250–270 g) and male rats (20–24 weeks) were housed in the same cages at a ratio of 2:1. During the breeding, female rats were examined daily by vaginal smear and were judged to be pregnant when vaginal plugs or sperm were found (gestational day, GD 0.5). Then, at GD 9.5, pregnant SD rats were divided into four groups according to their body weight in order to make sure that the average body weight in each group (around 280–290 g) was not significantly different.

The four groups are as follows: control group, L-NAME high-dose group (L-H), L-NAME low-dose group (L-L), and L-NAME low-dose + Coffee group (L-L + Coffee). The treatment for the animals in different groups is as follows: Control group (*n* = 8): oral gavage: water, intraperitoneal injection: saline; L-H (*n* = 11) and L-L (*n* = 7) groups: oral gavage: water, intraperitoneal injection: L-NAME at a concentration of 125 mg/kg and 10 mg/kg, respectively; L-L + Coffee group (*n* = 8): oral gavage: coffee, intraperitoneal injection: L-NAME at a concentration of 10 mg/kg. Coffee and L-NAME treatment was performed daily from GD10.5 to GD18.5. The coffee was given by oral gavage because humans drink coffee orally. Intraperitoneal injection was chosen for L-NAME administration according to literature.^(11)^

Pregnant rats in L-NAME low-dose + Coffee group were orally administered with the fresh coffee solution (1.8 ml/200 g) daily from GD 10.5 to GD 18.5. The coffee solution was brewed with Sinloy coffee powder according to the manufacturer’s instructions. The method we used to prepare coffee in this animal study is the same as that used to make coffee in daily life. The insoluble residues were filtered out. The coffee concentration was 3.86% (w/v), calculated by dividing the weight of the dried residue of brewed coffee solution by the volume of the coffee. The dose (1.8 ml/200 g body weight/d) given to rats is equivalent to 5 cups for humans according to the following calculation. The dose of 1 cup of coffee for one person per day in humans is approximately 125 mL/70 kg body weight/day, which is converted to 0.36 ml/200 g body weight/day in rats according to the body surface conversion. Therefore, the dose (1.8 mL/200g/d) given to rats in this study is approximately equivalent to 5 cups for humans. Although 5 cups of coffee per day are probably higher than average amount of coffee consumed by humans, due to the much shorter pregnancy time of rats compared to that of humans and the short exposure time in this study for only 8 d, this dose of coffee was used in the current study.

On GD 19.5, a cesarean section was performed on the pregnant rats, which were continuously anesthetised with isoflurane to minimise pain and discomfort. Non-pregnant female rats were excluded and only 34 female rats were left for analysis (Control group: *n* = 8, L-NAME high-dose group: *n* = 11, L-NAME low-dose group: *n* = 7, L-NAME low-dose + Coffee group: *n* = 8). The number, size and weight of fetuses and the number of stillbirths were recorded. The development of forelimbs and hind limbs and various organs were examined. The number and weight of placentas were recorded and measured.

### Blood pressure measurement

Systolic blood pressure (SBP) was measured in each rat at GD 9.5 and GD 18.5 using the tail-cuff plethysmography with Medlab biological signal acquisition system (Bilead, Shenzhen, China). After the pulse was stabilised, SBP was measured 3–4 times and the mean SBP was calculated.

### Urinary and serum parameters detection

Urine samples were collected by using metabolic cages on GD 9.5 and GD 18.5. The supernatant was collected after centrifugation at 3000 rpm for 10 min. Urinary albumin and creatinine were determined using a Thomas Brilliant Blue G-250 assay kit and a creatinine kit (Nanjing Jiancheng Bioengineering Institute, Nanjing, China), respectively using a microplate reader (BioTek Epoch, Vermont, USA). Serum isolated from blood samples was used to determine uric acid and creatinine (Nanjing Jiancheng Bioengineering Institute, Nanjing, China).

### Histology

The hematoxylin/eosin (HE) staining and immunohistochemical staining (IHC) were performed by Servicebio Technology Co., Ltd (Wuhan, China). The dissected tissue samples were fixed in formalin, and the fixed tissues were dehydrated and embedded with paraffin wax. The embedded placental tissues were cut into 5 μm thick wax slices in transverse and longitudinal directions, respectively. Staining was performed with HE. For IHC, sections were deparaffinised and rehydrated, and antigen was retrieved in 10 mM citrate buffer (pH 6.0). Sections were incubated with 3% hydrogen peroxide solution for inhibiting endogenous peroxidase. Then 3% BSA was used for blocking. The primary eNOS antibody (Servicebio, GB12086, 1:200) was used for IHC staining.

Microscope inspection and image acquisition were completed by a light microscope (CKX53; Olympius, Tokyo, Japan). For the quantitative analysis, we randomly selected 5 sections from each animal and 5 fields from each section. The software ImageJ (National Institutes of Health, Bethesda, Maryland, USA) was used for quantitative analysis.

### Statistical analysis

Each measurement was repeated three times. One-way ANOVA test was used. The unit of analysis is group of treatments. All data were presented as Mean ± Standard Deviation (SD). P < 0.05 was considered significant. Statistical analysis was performed using GraphPad Prism 8 software (GraphPad software, La Jolla, CA).

## Results

### Coffee causes defects in fetal development in pregnant rats treated with low-dose L-NAME

**Fig. 1.**
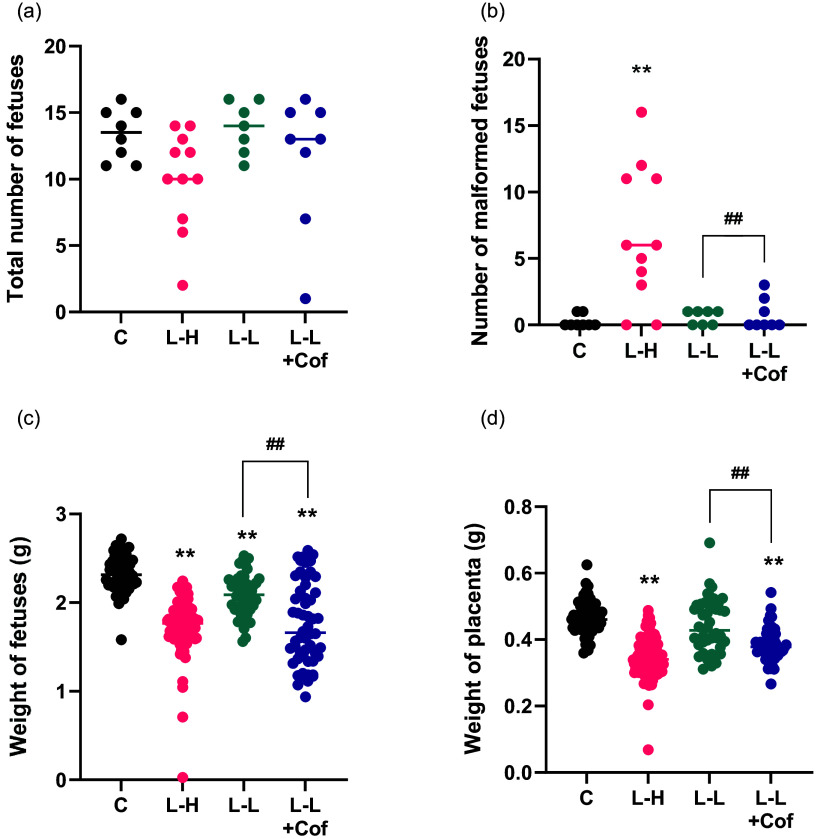
Fetus development in pregnant rats treated with L-NAME and/or Coffee. (a) Total number of fetuses, (b) number of malformation fetuses, (c) Weight of fetuses, (d) weight of placenta. C: Control group, L-H: L-NAME high-dose group, L-L: L-NAME low-dose group, L-L + Cof: L-NAME low-dose + Coffee group. Results are shown as mean ± SD. For (a) and (b), Control group: *n* = 8, L-NAME high-dose group: *n* = 11, L-NAME low-dose group: *n* = 7, L-NAME low-dose + Coffee group: *n* = 8; For (c) and (d), Control group: *n* = 52, L-NAME high-dose group: *n* = 96, L-NAME low-dose group: *n* = 44, L-NAME low-dose + Coffee group: *n* = 54., *P < 0.05; **P < 0.01 compared to control group; ^##^P < 0.01 compared between the two indicated groups.

Treatment with high-dose L‑NAME led to a significant reduction in placental and fetal weight and drastic increase in the number of malformed fetus relative to the control group (Fig. 1, Table 1). On the contrary, low-dose L-NAME caused only a modest decrease of fetus weight and no change in the placental weight and number of malformed fetus compared with the control group. However, when coffee was given together with low-dose L-NAME, the weight of fetus and placenta significantly decreased and the number of malformed fetus significantly increased compared to low-dose L-NAME group (Fig. 1, Table 1). The specific malformation of fetuses in L-NAME high-dose group and L-NAME low-dose + Coffee include subcutaneous stasis, hind limb hypoplasia and insufficient differentiation (Fig. S1).

**Table 1.** Comparison of fetus number, malformation rate, and death rate in each group

Group	Total number of fetuses	Malformation rate (%)	Death rate (%)
Control	107	1.87	0
L-NAME high dose	64	40.63	21.87
L-NAME low dose	96	4.17	1.04
L-NAME low dose + Coffee	92	6.52	9.78

### Coffee causes hypertension and proteinuria in pregnant rats treated with low-dose L-NAME

**Fig. 2.**
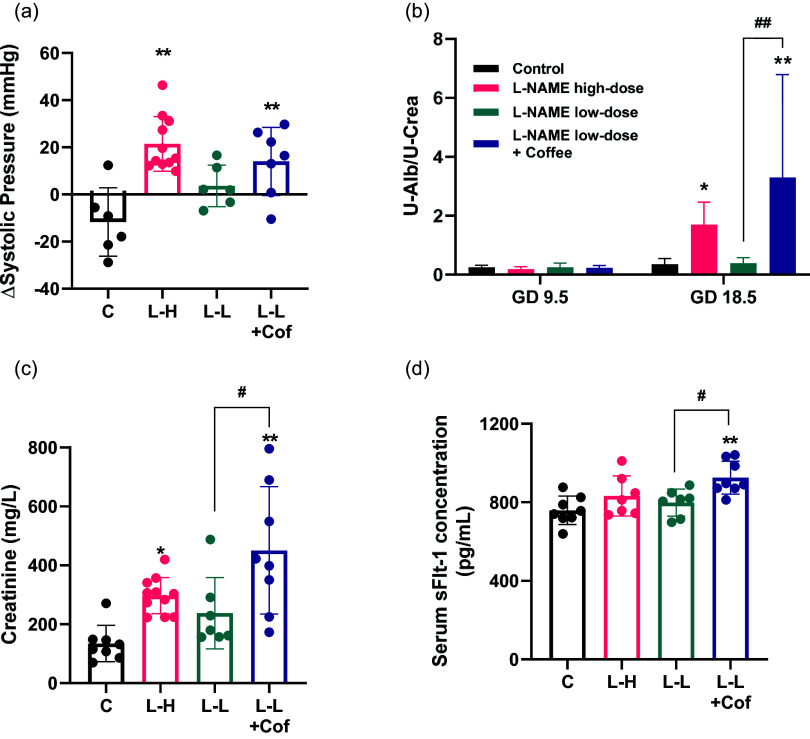
Systolic blood pressure (SBP) and renal functions of pregnant rats treated with L-NAME and/or Coffee. (a) The change in SBP on GD18.5 compared to GD9.5, (b) Urinary protein level indicated by urinary albumin (U-Ab)/urinary creatinine (U-Crea), (c) Serum creatine, (d) serum sFlt-1 level in pregnant rats. C: Control group, L-H: L-NAME high-dose group, L-L: L-NAME low-dose group, L-L + Cof: L-NAME low-dose + Coffee group. Results are shown as mean ± SD. Control group: *n* = 8, L-NAME high-dose group: n = 11, L-NAME low-dose group: *n* = 7, L-NAME low-dose + Coffee group: *n* = 8. *P < 0.05; **P < 0.01 compared to control group; ^#^P < 0.05; ^##^P < 0.01 compared between the two indicated groups.

SBP was measured on GD 9.5 (before treatment) and GD 18.5 (after treatment). On GD 9.5, SBP was not significantly different between each group. Compared with SBP at GD 9.5, SBP on GD18.5 in control group decreased by about 11 mmHg (Fig. 2a). On the contrast, SBP on GD18.5 in the high-dose L-NAME group increased by about 20 mmHg compared to that of GD9.5 (Fig. 2a). SBP on GD 18.5 in the low-dose L-NAME group remained unchanged compared to that of GD9.5 (Fig. 2a). The rats that received low-dose L-NAME + coffee had an increased SBP on GD18.5 by about 10 mmHg compared to that of GD9.5 (Fig. 2a). Overall, compared to control group, ΔSBP values in the high-dose L-NAME group and low-dose L-NAME + coffee group are significantly higher than that of control group (Fig. 2a). ΔSBP in the low-dose L-NAME + coffee group is higher than that of low-dose L-NAME group albeit without statistical significance due to large inter-individual variations (Fig. 2a).

The ratio of urinary albumin (U-Ab)/urinary creatinine (U-Crea) in the L-NAME high-dose group and the low-dose L-NAME + coffee group increased significantly after treatment compared with that of control group (Fig. 2b). On GD18.5, U-Ab/U-Crea in the low-dose L-NAME + coffee group increased significantly compared with that in the low-dose L-NAME group (Fig. 2b). In addition, there is a significant increase in creatinine content in serum in the L-NAME high-dose group and the low-dose L-NAME + coffee group (Fig. 2c). These results indicate that there is prominent kidney damage in the L-NAME high-dose group and the low-dose L-NAME + coffee group. The weight of the kidney and serum uric acid level was not significantly altered (Fig. S2).

Increased placental production of sFlt-1 is another important marker of PE. sFlt-1 level in the low-dose L-NAME + coffee group is significantly higher than that of control and low-dose L-NAME group (Fig. 2d). The high-dose L-NAME induced sFlt-1 level to some extent without statistical significance compared to control.

### Coffee causes prominent histological change in placenta in pregnant rats treated with low-dose L-NAME

**Fig. 3.**
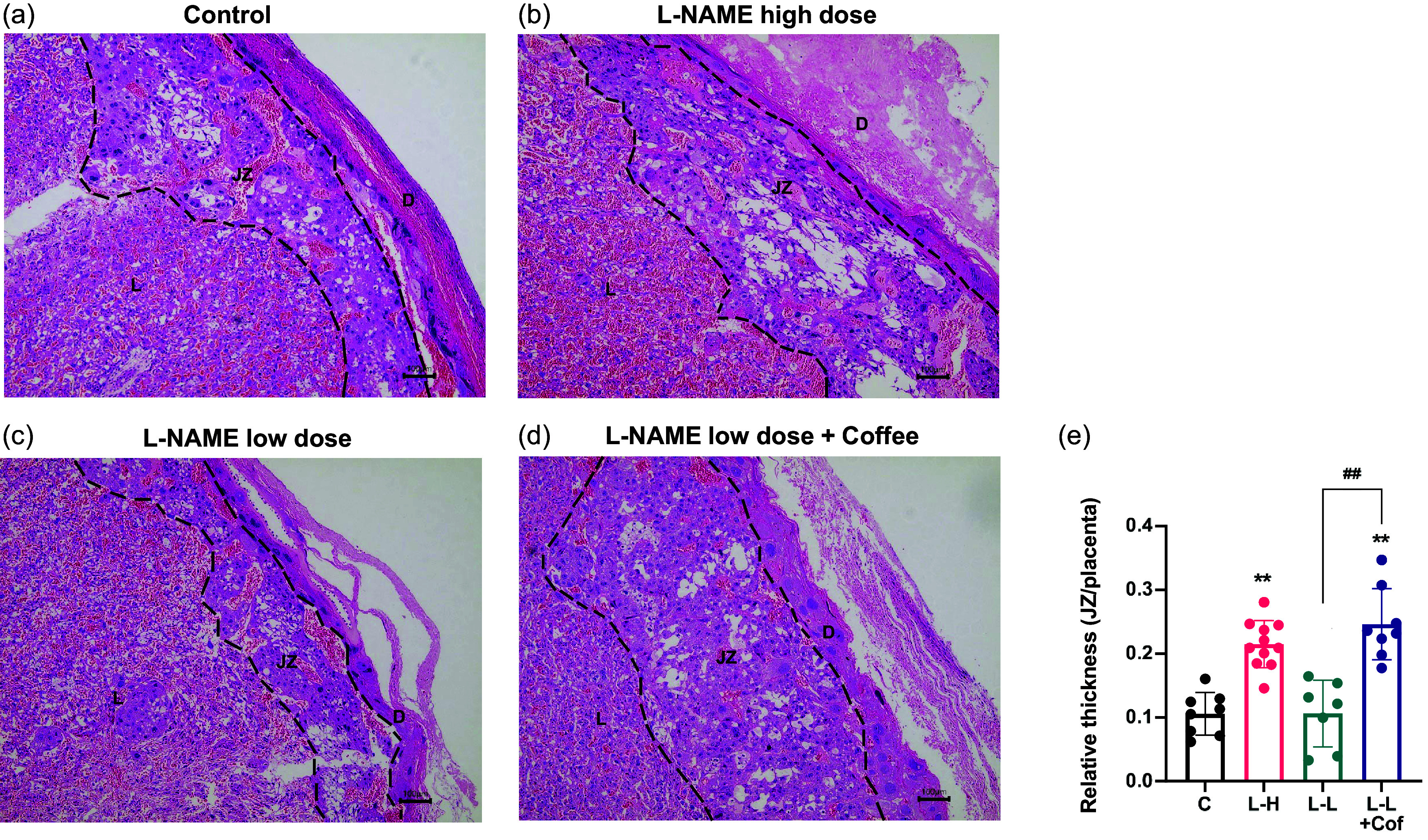
Changes of placenta structure in pregnant rats. Placental histology in hematoxylin/eosin-stained sections from the control group (a), the L-NAME high-dose group (b), the L-NAME low-dose group (c), and the L-NAME low-dose + Coffee group (d). The dotted line divides the placental structure into three parts. D, decidua, JZ, junctional zone; L, labyrinth. Scale bars: 100 μm. (e) The relative thickness of the junctional zone (JZ). Results are shown as mean ± SD. Control group: *n* = 8, L-NAME high-dose group: *n* = 11, L-NAME low-dose group: *n* = 7, L-NAME low-dose + Coffee group: *n* = 8. **P < 0.01 compared to control group; ^##^P < 0.01 compared between the two indicated groups.

Histological analysis of the placental tissue was carried out to probe the tissue damage in the placenta. The placenta can be divided into the decidua layer, junctional zone, and labyrinth zone in the cross section (Fig. 3). There is a significantly thickened junctional zone in the placenta of L-NAME high-dose group and the low-dose L-NAME + coffee group compared to the control group (Fig. 3). The junctional zone in the placenta of the low-dose L-NAME + coffee group is significantly thicker compared to the low-dose L-NAME group (Fig. 3).

**Fig. 4.**
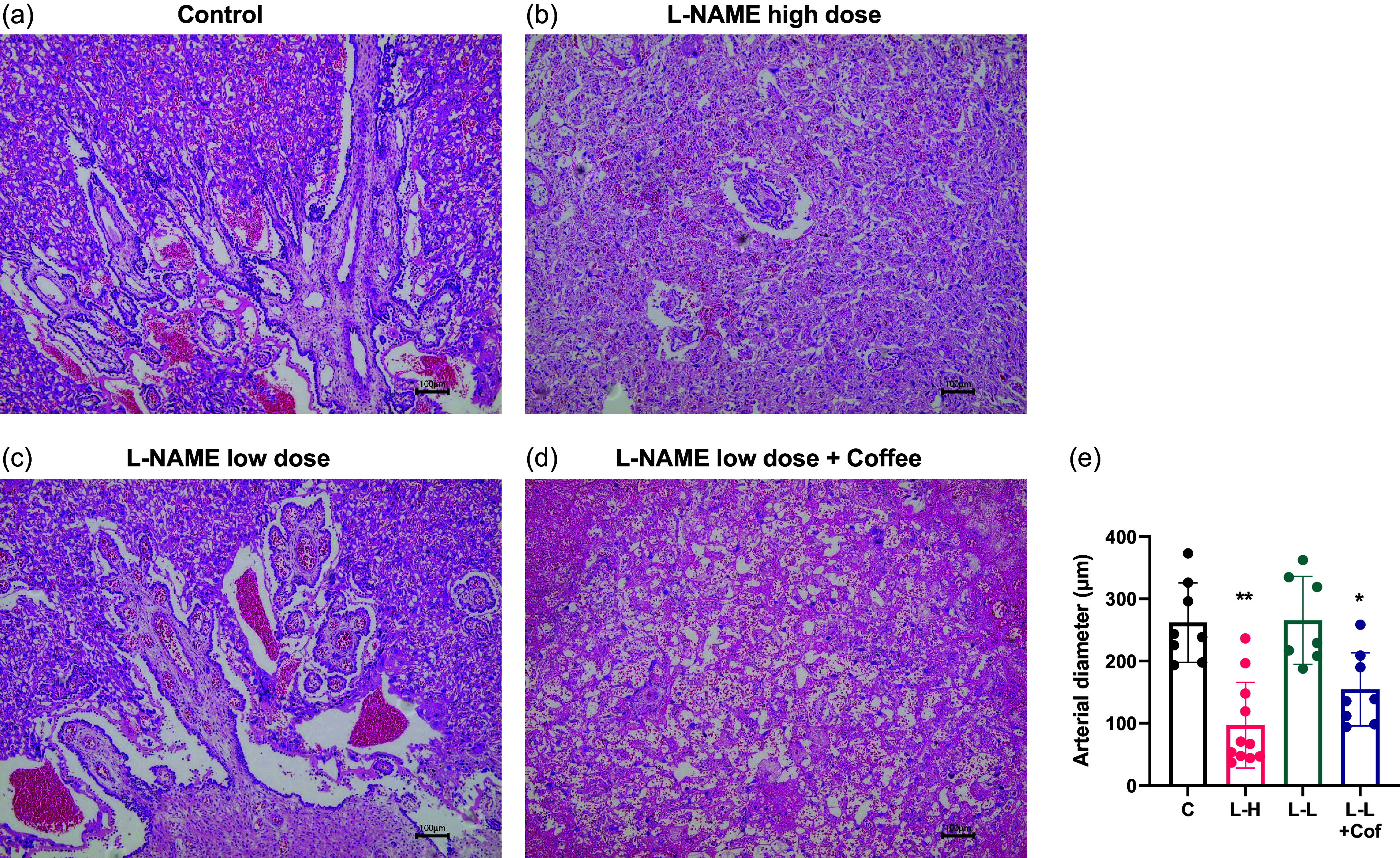
Comparison of vascular network distribution in placenta. Cross sections of labyrinth region of placenta in the control group (a), the L-NAME high-dose group (b), the L-NAME low-dose group (c), and the L-NAME low-dose + Coffee group (d). Scale bars: 100 μm. (e) The arterial diameter in the labyrinth region. Results are shown as mean ± SD. Control group: *n* = 8, L-NAME high-dose group: *n* = 11, L-NAME low-dose group: *n* = 7, L-NAME low-dose + Coffee group: *n* = 8. *P < 0.05; **P < 0.01 compared to control group.

We next examined the labyrinth region of the placenta with longitudinal section (Fig. 4). Compared with control group, placentas from L-NAME high-dose group and the low-dose L-NAME + coffee group had poorly development vasculature and arterial diameters were significantly smaller (Fig. 4e). In the low-dose L-NAME group, vasculature structure in placenta was generally normal.

**Fig. 5.**
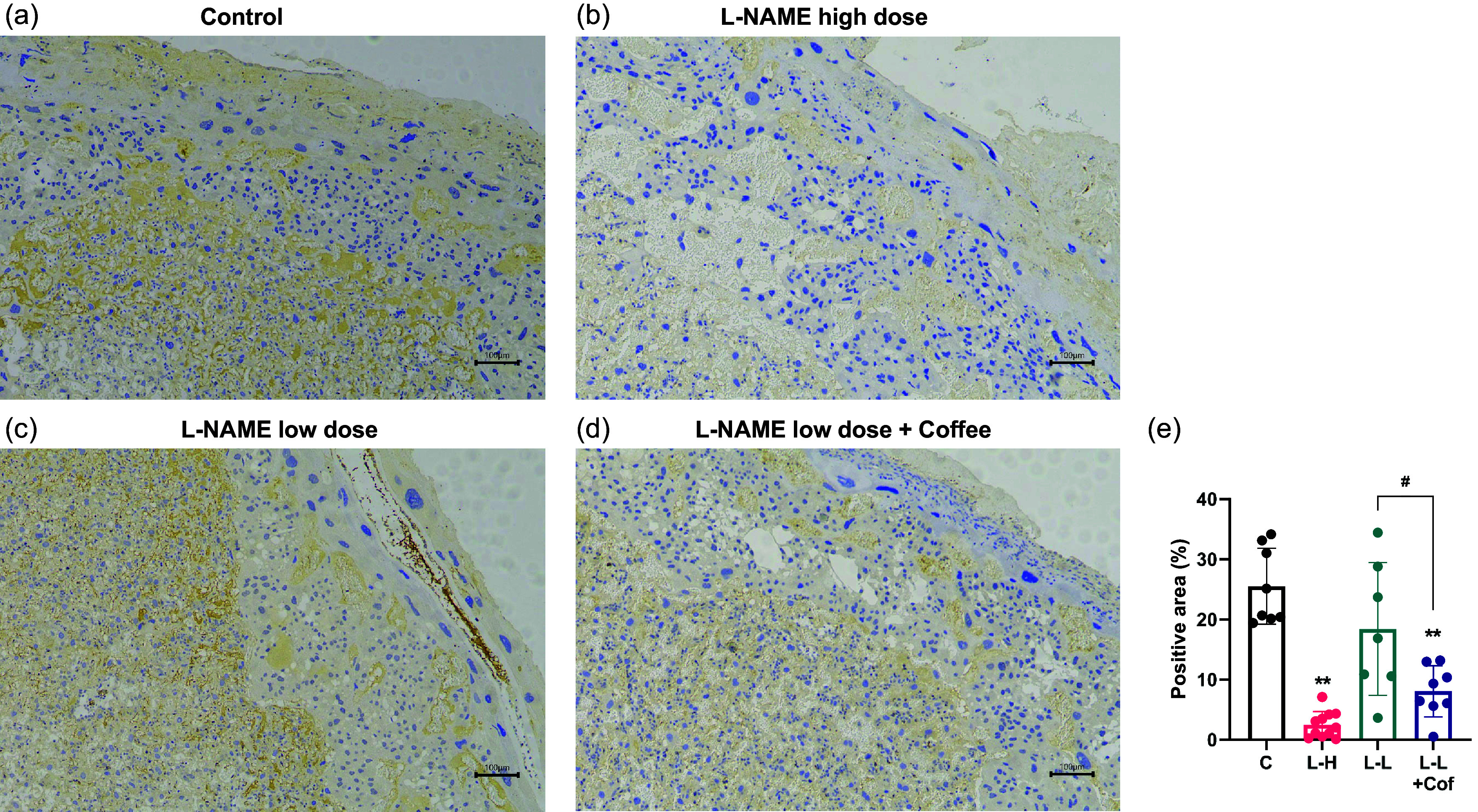
Comparison of eNOS expression in placenta. Immunohistochemical staining of placenta with eNOS antibody in the control group (a), the L-NAME high-dose group (b), the L-NAME low-dose group (c), and the L-NAME low-dose + Coffee group (d), Scale bar =100 μm. (e) The quantitative analysis of the positive area (%). Results are shown as mean ± SD. Control group: *n* = 8, L-NAME high-dose group: *n* = 11, L-NAME low-dose group: *n* = 7, L-NAME low-dose + Coffee group: *n* = 8. *P < 0.05; **P < 0.01 compared to control group, ^#^P < 0.05 compared between the two indicated groups.

eNOS is important for synthesis of nitric oxide (NO), a vessel dilator. Expression of eNOS significantly decreased in the placenta in the L-NAME high-dose group and the low-dose L-NAME + coffee compared to control, but not in low-dose L-NAME group (Fig. 5).

## Discussion

There has been growing concerns in recent years about the potential health risk of coffee consumption during pregnancy.^(12,13)^ L-NAME is a NOS inhibitor, which can inhibit the activity of NOS in human placental trophoblast cells and reduce the production of NO.^(14,15)^ It is a widely used tool compound for inducing PE in animals,^(10)^ because vascular endothelial NO has been reported to be a vital factor to protect vascular damage.^(16)^ We used a commonly used dose of L-NAME (125 mg/kg) to establish a positive PE model and a low dose of L-NAME (10 mg/kg) to simulate some pregnant women who have a predisposed vascular problem. We studied whether coffee intake during pregnancy was sufficient to induce PE in these susceptible populations. Our results show that coffee intake during pregnancy can lead to the symptoms of PE such as fetal growth restriction, hypertension, proteinuria, and fetal growth in low-dose L-NAME treated animals.

Pregnancy induces extensive adaptations in cardiovascular physiology. NO production by eNOS has been found to be important for the maintenance of normal blood pressure.^(17)^ We found that eNOS expression significantly decreased in the placenta in the L-NAME high-dose group and the low-dose L-NAME + coffee compared to control, which suggests that the reduced production of NO in placenta may contribute to the high blood pressure seen in the L-NAME high-dose group and the low-dose L-NAME + coffee group. Further investigations are merited to study the mechanism of the downregulation of eNOS by coffee.

Proteinuria is not essential to diagnosis but is related to disease severity and fetal outcomes in PE patients.^(1)^ We found that urinary albumin levels increased in L-NAME high-dose group and L-NAME low-dose + coffee group (Fig. 2b). Multiple studies suggest that long-term administration of L-NAME alters various biochemical markers of the kidney by inhibiting NOS, which is consistent with our result.^(18,19)^ However, at present, there are few clinical studies on the effect of coffee on the risk of kidney injury during pregnancy, which merits further investigation.

The histology of placenta in L-NAME high-dose group and L-NAME low-dose + coffee group showed that the thickness of the junctional zone increased compared to control and L-NAME low-dose group (Fig. 3), and the vasculature in the labyrinth region was poorly developed with smaller arterial diameters (Fig. 4). sFlt-1 is induced by hypoxia inducible factor-1 and is a marker of vascular endothelium dysfunction.^(20)^ Elevated sFlt-1 level also may lead to the decrease of placental vascular development, thus affecting fetal nutrition and oxygen supply, and causing fetal growth restriction.^(21)^ In addition, NO and eNOS play important roles in placental vasculature maturation.^(22)^ Therefore, elevated sFlt-1 and decreased eNOS induced by L-NAME high-dose treatment and L-NAME low-dose + coffee treatment may contribute to the impaired placenta structure and function in these two groups.

It is of note that the malformations of fetuses were slightly different between the high-dose L-NAME group and the L-NAME low-dose + coffee group. In the high-dose L-NAME group, hind limb developmental defects characterised by severe hemorrhage were dominant, while subcutaneous stasis was the dominant malformations in the L-NAME low-dose + coffee group. This may be due to their different mechanisms of inducing PE symptoms. Reports have revealed NO production controls late embryonic hind limb development.^(15)^ Therefore, high-dose L-NAME treatment caused hind limb defects. Subcutaneous bleeding in the L-NAME low-dose + coffee group may be related to hormonal disruption caused by coffee. There were also cases of forelimb defect in the L-NAME low-dose + coffee group, which should have been differentiated on GD12.5.^(23)^ One plausible hypothesis is that it could be attributed to the impaired ectodermal cristae and active polarisation centre, which play a crucial role in the development of limb buds.^(24)^ The mechanisms for these differences in fetus development merit further investigation.

In this study, we have found that coffee consumption during pregnancy with underlying PE risk can induce symptoms of PE in animals. Further clinical studies are merited to validate this finding in humans. In addition, there are lots of components in coffee such as various coffee polyphenols. Further studies are merits to determine whether caffeine or a particular coffee polyphenol could induce PE in pregnant rats and the underlying mechanisms.

In conclusion, our findings reveal the potential for the increased PE risk by coffee intake during pregnancy in pregnant individuals with underlying vasculature problems. Therefore, coffee intake during pregnancy could be potentially hazardous for the health of both mother and fetus.

## Supporting information

Chen et al. supplementary material Chen et al. supplementary material

## Abbreviations

**ELISA:** enzyme-linked immunosorbent assay; **eNOS:** endothelial nitric oxide synthase; **GD:** gestational day; **HE:** hematoxylin/eosin; **IHC:** immunohistochemical; **L-NAME:** NG-nitro-L-arginine methyl ester; **NO:** nitric oxide; **NOS:** nitric oxide synthase; **PE:** Preeclampsia; **SBP:** Systolic blood pressure; **SD:** Sprague-Dawley; **sFlt-1:** soluble fms-like tyrosine kinase-1; **U-Ab:** urinary albumin; **U-Crea:** urinary creatinine.

## Supplementary material

The supplementary material for this article can be found at https://doi.org/10.1017/jns.2024.36
