# Supplementary material for: Coffee intake leads to preeclampsia-like syndromes in susceptible pregnant rats
Source: J Nutr Sci. 2024 Sep 18;13:e34. doi: 10.1017/jns.2024.36 (PMC11418084; doi:10.1017/jns.2024.36)
Supplement: Chen et al. supplementary material [file S2048679024000363sup001.pdf]

Supplementary data

Figure legend

Fig. S1. Representative images of fetuses from each group. Red circle: hind limb problem; Blue rectangle: dead fetus.

Fig. S2. Change in uric acid, kidney weight in pregnant rats. Serum uric acid (A), kidney weight (B) of pregnant rats in each group. C: Control group, L-H: L-NAME high dose group, L-L: L-NAME low dose group, L-L + Cof: L-NAME low dose + Coffee group. Results are shown as mean ± SD. Control group: n = 8, L-NAME high dose group: n = 11, L-NAME low dose group: n = 7, L-NAME low dose + Coffee group: n = 8. *P < 0.05; **P < 0.01 compared to control group.

Figure S1

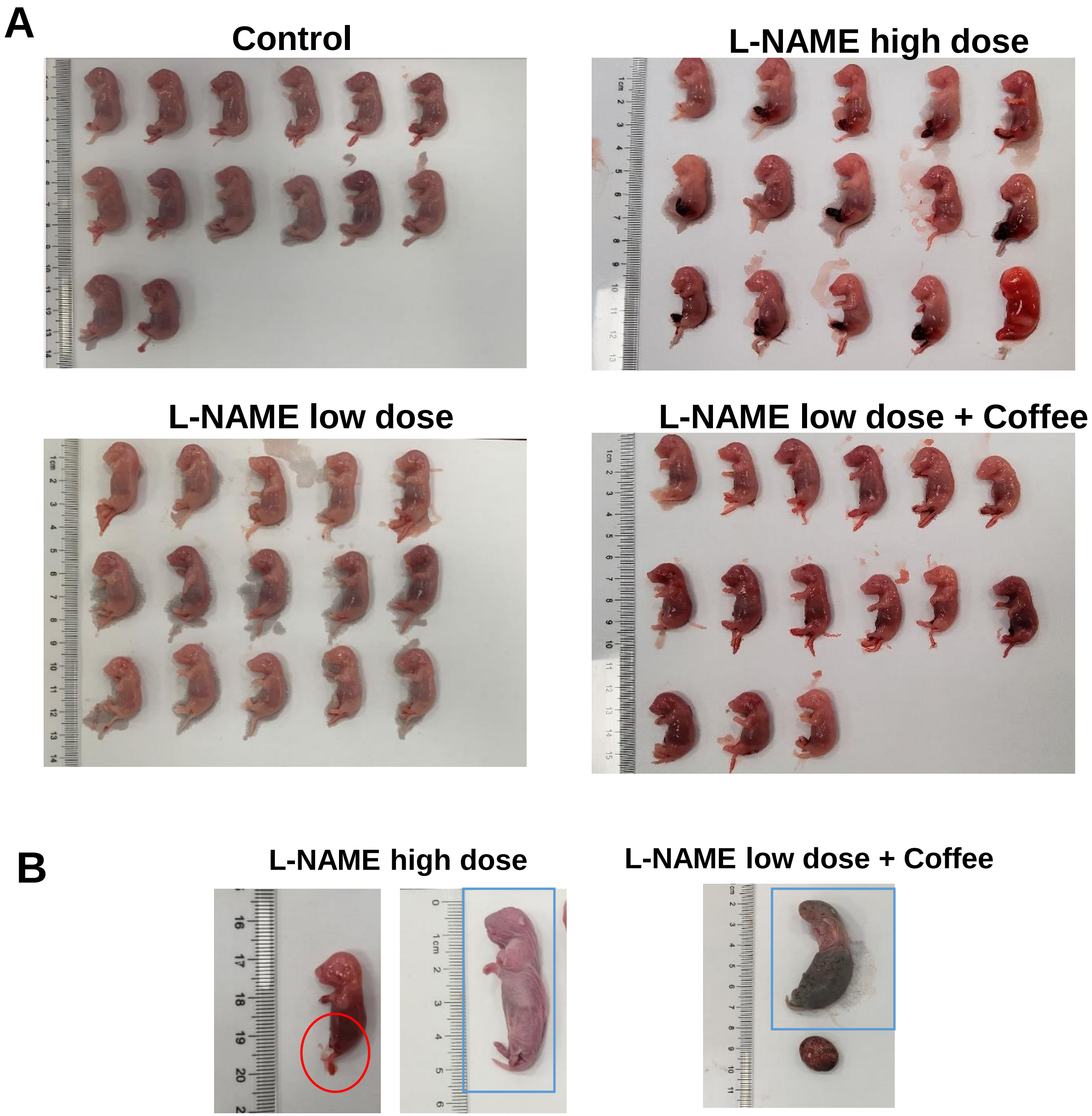
A
Control
L-NAME high dose
L-NAME low dose
L-NAME low dose + Coffee
B
L-NAME high dose
L-NAME low dose + Coffee

Figure S2

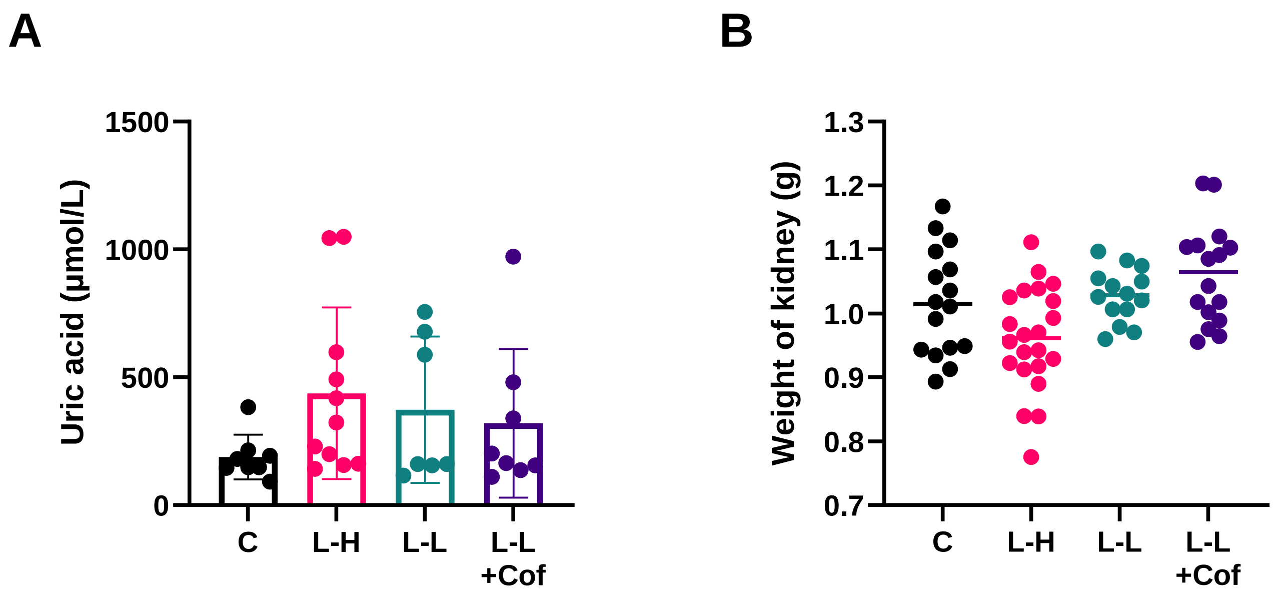
A
B
Uric acid (μmol/L)
1500
1000
500
0
C
L-H
L-L
L-L
+Cof
Weight of kidney (g)
1.3
1.2
1.1
1.0
0.9
0.8
0.7
C
L-H
L-L
L-L
+Cof
